# Retraction: Grey relational analysis method for typhoon vulnerability assessment of civil engineering structures based on the 2-tuple linguistic neutrosophic number

**DOI:** 10.1371/journal.pone.0286615

**Published:** 2023-05-30

**Authors:** The PLOS ONE Editors

After this article was published, similarities were noted between this article and submissions by other research groups which call into question the validity and provenance of the reported results, and the adherence of this article to the PLOS Authorship policy. Further editorial assessment identified concerns regarding the integrity of the peer review process. In light of these issues, the *PLOS ONE* Editors retract this article [1].

FW did not agree with the retraction. All other authors either did not respond directly or could not be reached.

## References

[1] Qi Y, Zhu C, Wang F, Xia Y (2022) Grey relational analysis method for typhoon vulnerability assessment of civil engineering structures based on the 2-tuple linguistic neutrosophic number. PLoS ONE 17(11): e0277539. 10.1371/journal.pone.0277539 doi: 10.1371/journal.pone.0277539 36378666 PMC9665396

